# Impact of Organic Anions on Metal Hydroxide Oxygen Evolution Catalysts

**DOI:** 10.1021/acscatal.4c01907

**Published:** 2024-07-29

**Authors:** Shujin Hou, Lili Xu, Soumya Mukherjee, Jian Zhou, Kun-Ting Song, Zhenyu Zhou, Shengli Zhang, Xiaoxin Ma, Julien Warnan, Aliaksandr S. Bandarenka, Roland A. Fischer

**Affiliations:** † Physics of Energy Conversion and Storage, School of Natural Sciences, Department of Physics, Technical University of Munich, James-Franck-Straße 1, Garching 85748, Germany; ‡ Inorganic and Metal−Organic Chemistry, School of Natural Sciences, Department of Chemistry, Technical University of Munich, Lichtenbergstraße 4, Garching 85748, Germany; § Department of Chemistry and Biochemistry and the Oregon Center for Electrochemistry, University of Oregon, Eugene, Oregon 97403, United States; ∥ Department of Chemical & Biomolecular Engineering, University of California, Berkeley, California 94720, United States; ⊥ Institute of Optoelectronics & Nanomaterials, College of Materials Science and Engineering, Nanjing University of Science and Technology, Nanjing, Jiangsu 210094, China; # Bernal Institute, Department of Chemical Sciences, University of Limerick, Limerick V94 T9PX, Ireland; ¶ School of Chemistry and Chemical Engineering, Nanchang University, Nanchang 330031, P. R. China; ∇ Catalysis Research Center, Technical University of Munich, Ernst-Otto-Fischer-Straße 1, Garching 85748, Germany

**Keywords:** electrocatalysis, oxygen evolution reaction, metal−organic frameworks, metal hydroxides, organic anions

## Abstract

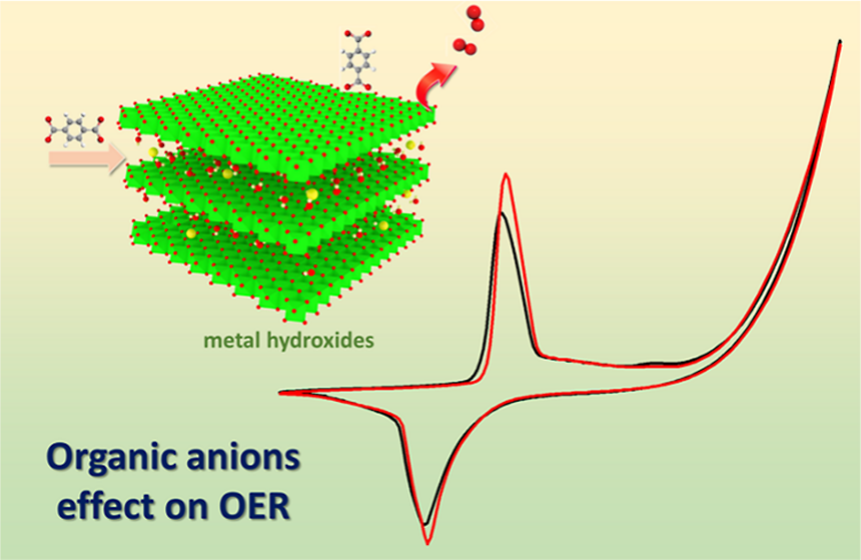

Structural metamorphosis of metal–organic frameworks (MOFs) eliciting highly active metal-hydroxide catalysts has come to the fore lately, with much promise. However, the role of organic ligands leaching into electrolytes during alkaline hydrolysis remains unclear. Here, we elucidate the influence of organic carboxylate anions on a family of Ni or NiFe-based hydroxide type catalysts during the oxygen evolution reaction. After excluding interfering variables, i.e., electrolyte purity, Ohmic loss, and electrolyte pH, the experimental results indicate that adding organic anions to the electrolyte profoundly impacts the redox potential of the Ni species versus with only a negligible effect on the oxygen evolution activities. In-depth studies demonstrate plausible reasons behind those observations and allude to far-reaching implications in controlling electrocatalysis in MOFs, mainly where compositional modularity entails fine-tuning organic anions.

## Introduction

Renewable electricity, e.g., wind and solar, producing green hydrogen energy is seen as a powerful way forward to support sustainable, carbon-neutral goals.^1−3^ However, electrochemical water splitting currently suffers from the slow kinetics of oxygen evolution reaction (OER).^4^ Developing facile approaches to enhancing OER catalytic activity by lowering activation barriers at the electrode/electrolyte interfaces is essential.^5−7^

Over the past decade, metal–organic framework (MOF)-based electrocatalysts have surged, translating from the discovery of conductive MOFs and two-dimensional MOFs to the study of catalytic mechanisms. To that end, multiple research groups have been increasingly probing several catalytic reactions in MOFs, whose mechanisms are often intertwined.^8−10^ It follows that understanding catalysis mechanisms represents a pressing challenge, including bespoke studies of yet unknown effects therein. Our groups reported earlier the facile synthesis of highly active OER electrocatalysts by *in situ* structural transformation and self-activation of surface-mounted MOFs (SURMOFs).^11−13^ Our more recent studies^14,15^ point to a correlation between MOF composition (especially ligand species) and catalyst stability, which in turn affects the mechanism of reconstruction or metamorphosis of the active species under OER conditions. These studies revealed the electrocatalytically active sites in several SURMOF derivatives and that reconstitution is a synergy of ligand dissociation and metal hydroxide formation. Presumptively, one would first draw attention to the reconstruction mechanism and the derived catalyst. However, the role of organic ligands after leaching into the electrolyte has rarely been studied.^16,17^ In fact, according to a vast number of studies concerning the effect of inorganic anions on OER catalysts,^18−20^ the organic ligand anions in the electrolyte should also act as a key factor. It is therefore urgent to elucidate their impacts and the factors underpinning them.

Thanks to recent insights into this matter,^11^ Ni–Fe bimetallic hydroxides are known to deliver benchmark OER catalytic activity. In this work, we investigate the effect of organic anions, namely, carboxylates, upon electrocatalysis using prototypical reference systems, i.e., the electrodeposited Ni(OH)_2_ and NiFe hydroxides. Electrolyte purity, ohmic loss, and electrolyte pH dependence were studied sequentially to control the systems. Further, a range of complementary techniques, such as *in situ* Raman, fourier-transform infrared spectroscopy (FT-IR), grazing incidence X-ray diffraction (GIXRD), and density functional theory (DFT) calculations, were conducted in detail.

## Results and Discussion

**Figure 1:**
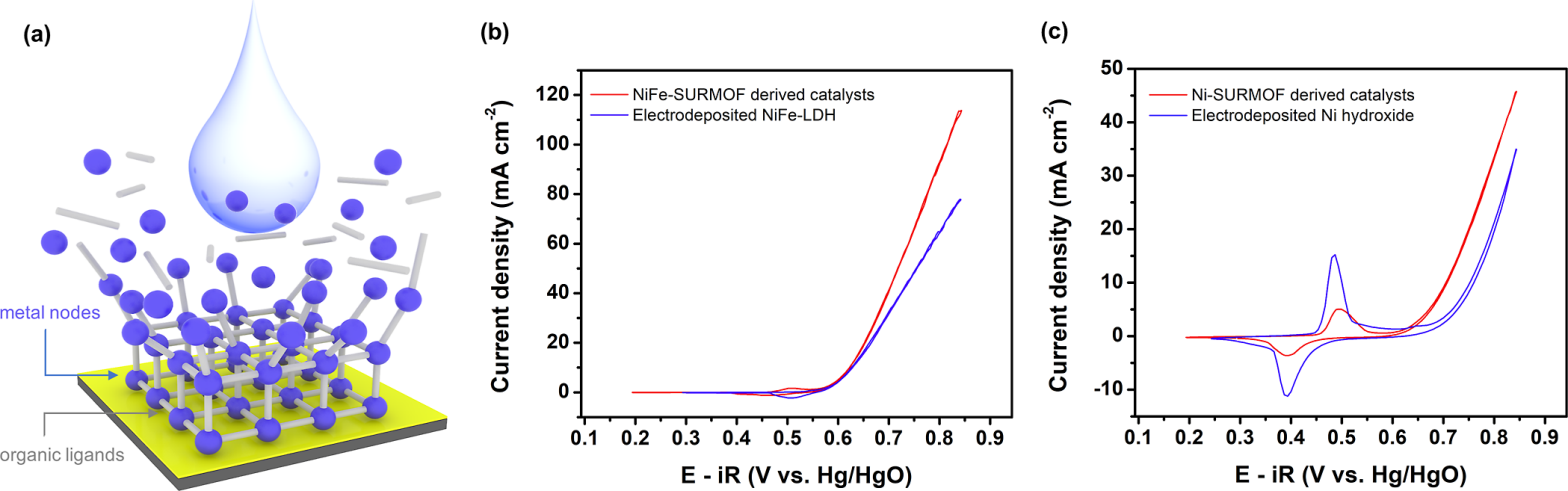
(a) Schematic illustration of alkali-unstable MOFs. Comparison of the OER activities for (b) NiFe-SURMOF derived catalyst and electrodeposited NiFe-layered double hydroxide (NiFe-LDH) as well as (c) Ni-SURMOF derived catalyst and electrodeposited Ni-hydroxide. Cyclic voltammetry (CV) curves were recorded after the third cycle (stable CV).

NiFe- or Ni-based SURMOFs were prepared, which is consistent with the literature on layer-by-layer methods.^11,13^ As a result of the structural transformations, alkali-unstable SURMOFs were found to be converted into amorphous metal hydroxides accompanied by the alkaline leaching of organic ligands (Figure 1a). The subsequent electrochemical activation was found to optimize the metal hydroxide structures further, exposing more active sites.^11,21^ To illustrate the merits of SURMOF evolution as a catalyst design paradigm, traditional electrodeposition was employed to prepare NiFe-LDHs and Ni-hydroxide as reference systems (Figure S1). The SURMOF-derived electrocatalysts revealed higher OER activities than the deposited catalyst films, as Figure 1b,c reveal. To circumvent any interference from catalyst loading differences on performance assessment, charge-normalized polarization curves were recorded on the Ni-SURMOF derived catalyst and Ni-hydroxide by integrating the oxidation peaks of Ni species (Figure S2). The observations can be ascribed to the crystalline–amorphous phase transformation and the highly electroactive surface areas found in the ensuing derivatives, which align with our previous work.^11^

**Figure 2:**
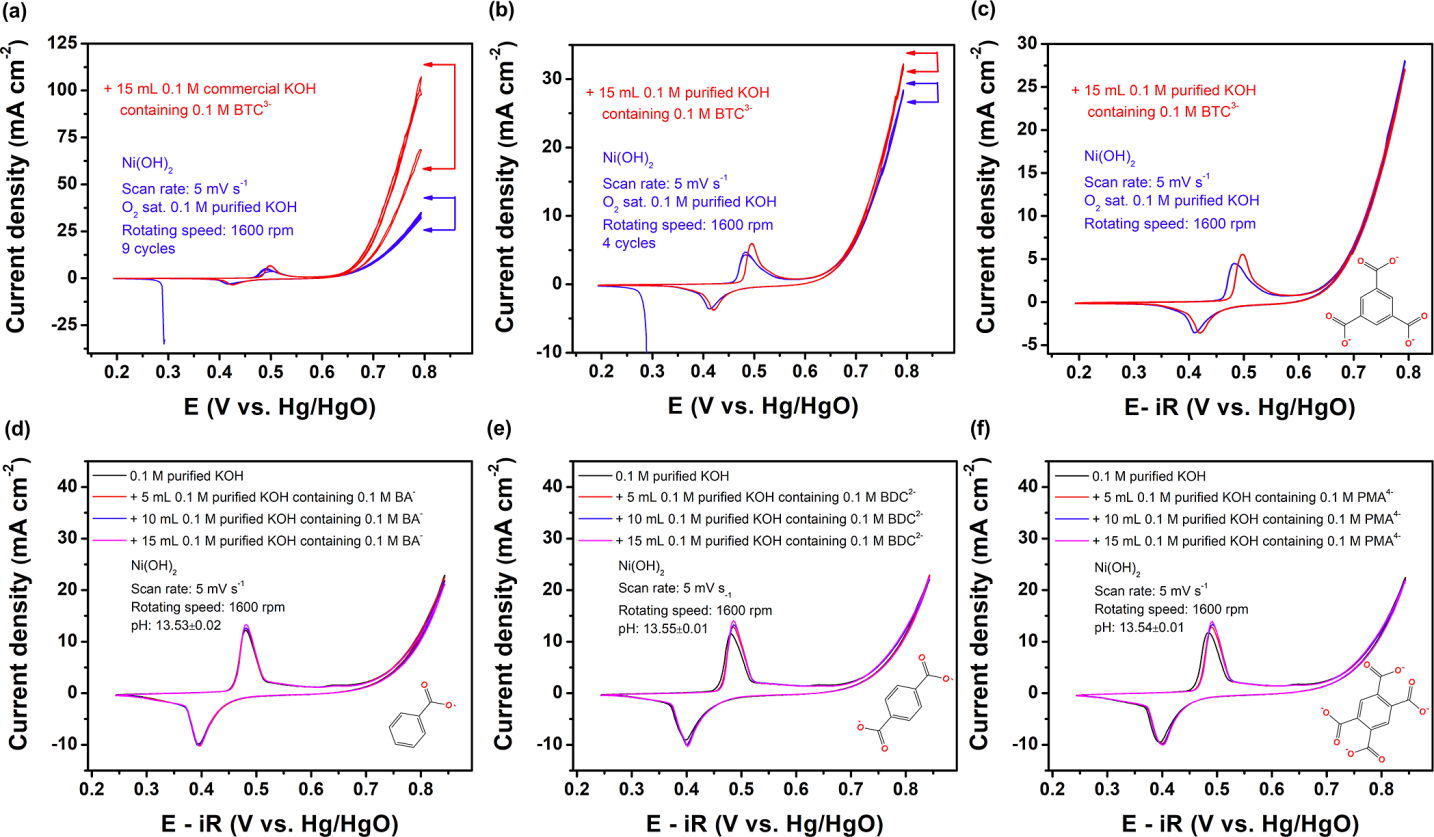
(a) CV curves of Ni(OH)_2_ in 0.1 M purified KOH solution and after the addition of 15 mL of 0.1 M commercial KOH containing 0.1 M BTC^3–^. (b) CV curves of Ni(OH)_2_ before and after the addition of BTC^3–^ to a 0.1 M purified KOH solution. (c) *iR* compensated CV curves before and after the addition of BTC^3–^. (d–f) Variations of CV curves with the addition of different amounts of BA^–^, BDC^2–^, and PMA^4–^. All experiments were performed in an O_2_-saturated basic electrolyte.

Despite high electrocatalytic performances, the role of dissolved organic ligands in the reconstruction process remains unknown thus far. SURMOF transformation entails an intricate process that comprises (a) metal–ligand bond cleavage, (b) recombination rules applying to metal nodes/clusters in their respective speciations, (c) lability/stability of the ligands, (d) electrochemical activation effects, among other things.^22−24^ Thanks to the detection of metal hydroxides as the (SUR)MOF derivatives,^11,23,25^ experiments aiming to unmask the ligand effect can be designed by using Ni- or NiFe-hydroxides as reference systems. In this context, carboxylate anions introduced in the electrolyte are regarded as the only variable in the following studies and discussion thereof. As shown in Figure S3a, before testing the OER properties, the electrodeposited Ni(OH)_2_ thin films were electrochemically activated at a sweep rate of 20 mV s^–1^ to obtain a steady-state chemical structure. However, the catalyst could still be activated upon increasing the voltage to 0.693 V vs Hg/HgO. This was supported by a gradual increase of the OER current over increasing scans, as Figure S3b demonstrates. After 30 cycles, 15 mL of 0.1 M BDC^2–^/KOH (H_2_BDC, benzene-1,4-dicarboxylic acid; 0.1 M BDC^2–^/KOH represents a solution containing 0.1 M BDC^2–^ and ∼0.1 M OH^–^) was added to the electrolyte. A significant current increase was observed, indicative of the role played by additional carboxylates in enhancing the activity. Five-hour CV over the deposited Ni(OH)_2_ was performed in the deuterated BDC^2–^/KOD electrolyte to rule out the electrochemical instability of the organic ligand, and the nuclear magnetic resonance (NMR) results specify that the BDC ligand is stable in the above experiments (Figure S3c). Nevertheless, the activity of Ni catalysts is known to be sensitive to electrolyte impurities, especially that of trace iron.^6,26^ In this activation process, 0.1 M KOH was prepared using commercial KOH pellets [impurities of iron, 0.87 μmol L^–1^ in 1.0 mol L^–1^ KOH were confirmed by inductively coupled plasma-optical emission spectroscopy (ICP-OES)]. To exclude the effect of iron, the KOH solution was prepurified following literature,^26^ denoted after this as purified KOH (impurities of iron, 0.09 μmol L^–1^ in 1.0 mol L^–1^ KOH determined by ICP-OES). The activation curve for freshly prepared Ni(OH)_2_ exhibits smaller redox couple shifts than that in the commercial KOH solution (Figure S4). In fact, incorporating iron from the electrolyte enables the redox peaks of Ni(OH)_2_/NiOOH to shift positively since the charge effect between Ni and Fe raises the difficulty of oxidizing Ni^2+^ to Ni^3+/4+^.^27,28^ After 9 activation cycles, the OER polarization curves attained a stable state, as indicated in Figure 2a. However, the OER current density dramatically increases after addition of deprotonated H_3_BTC (benzene-1,3,5-tricarboxylic acid). Despite the subtle differences between BDC^2–^ and BTC^3–^, the enhanced activity is understood to stem from the added carboxylic acid component. It is worth noting that BTC^3–^ was dissolved in commercial KOH; thus, the effect of trace iron was inevitable. In Figure 2b, an analogous experiment was conducted, but H_3_BTC was dissolved in purified KOH. A slight OER current increase was observed at high potential when BTC^3–^ was found to be present in the electrolyte. Introducing charged ions into the electrolyte will likely increase the conductivity proportionately. To accurately determine the influence of organic ligands as anions, the *iR* compensation of the polarization curve was analyzed, excluding the ohmic loss. Interestingly, as Figure 2c suggests, the introduction of BTC^3–^ is found to have no effect on the OER performance, by taking both electrolyte purity and *iR* compensation into consideration. Possible effects from three other carboxylate anions, benzoate (BA^–^), pyromellitate (PMA^4–^), and 2,3,5,6-tetrafluoro-1,4-benzenedicarboxylate (TFBDC^2–^) were investigated in this work. Solutions containing each of these were added to the electrolyte in batches, none of which caused an increase in the OER activity (Figures 2d–f, S5 and S6a). The electrolyte pH was maintained constant before and after introducing these carboxylates. The aim was to ensure that adding organic acids did not elicit any pH fluctuation in the bulk electrolyte. Despite indifference to the OER performances of the Ni hydroxide catalysts, the addition of carboxylates significantly altered the Ni species’ redox couple potentials.

Further, the effect of carboxylate ligands on NiFe-LDH is investigated (Figure S7). The results show no influence on the OER activity, with additional BDC^2–^ and BTC^3–^ remaining dormant in the solution. These results indicate the negligible impact of organic anions on the intrinsic oxygen evolution activity of Ni/Fe-based metal hydroxides.

**Figure 3:**
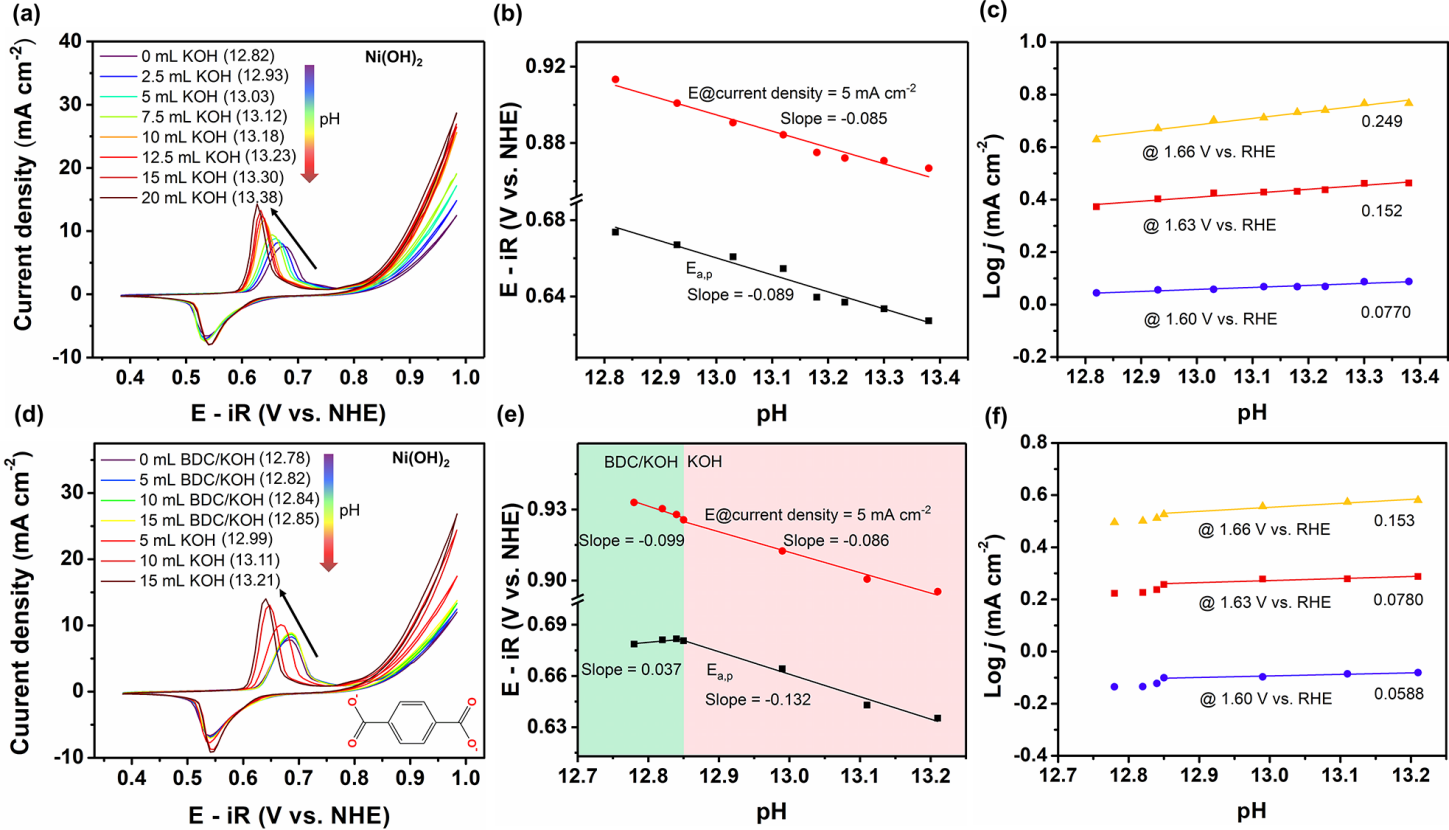
(a) pH-dependent CV curves of Ni(OH)_2_. The initial electrolyte was prepared by mixing 10 mL of 1 M purified KOH and 190 mL of water. Here, we define it as 0 mL KOH, followed by the addition of different volumes of 1 M purified KOH. All figures refer to the cumulative volume of KOH added afterward. (b) As a function of pH, the anodic oxidation peaks (*E*_a,p_) and potentials were obtained at the current density of 5 mA cm^–2^. (c) Reaction order of pH values with the OER activities at 1.60, 1.63, and 1.66 V versus the reversible hydrogen electrode (RHE). (d) pH-dependent CV curves of Ni(OH)_2_ in the presence of BDC^2–^. BDC/KOH represents a solution containing 0.1 M BDC^2–^ and 0.1 M KOH. (e) Both *E*_a,p_ and potentials obtained at the current density of 5 mA cm^–2^ as a function of pH. The pH dependence includes both BDC/KOH and KOH effects. (f) Reaction order of pH values with the OER activities at 1.60, 1.63, and 1.66 V vs RHE in the presence of BDC^2–^. The potentials at the current density of 5 mA cm^–2^ (b,e) and all current densities in (c,f) were recorded based on the cathodic polarization curves.

Taking cognizance of the redox peak potential shifts by adding organic anions, the plausible reasons for this observation were investigated. All oxidation peak potential shifts were extracted for comparison (Figure S8). The potential shifts plotted versus the number of carboxyl groups (found in the organic ligands) demonstrate a linear trend. The more negatively charged BTC^3–^ reveals a larger positive potential shift (∼16 mV), suggesting that introducing organic anions can significantly affect the kinetics of nickel oxidation reaction while contributing minimally to the catalytic activity.^26^ The oxidation peak potential shifts exhibit a linear relationship with the first dissociation constants (p*K*_1_) of the organic acids (Figure S8b).^29^ The discrepancy in p*K*_1_ values influences the local pH where the organic anions are located. Naturally, it is reasonable to assume that the redox peak shift is directly related to the local pH or proton transfer capacity.^30,31^ More explanations of using the inductive effect refer to Supporting Information. Studying the effect of pH upon oxidation potential shift and OER activity is crucial for understanding the roles of (a) organic anions and (b) reaction kinetics.^32^ pH-dependent experiments were conducted in strongly alkaline electrolytes, as shown in Figure 3. Extracting data from CV curves at different pHs enables us to determine the anodic oxidation peaks (*E*_a,p_) and the potentials registered under a specific current density of 5 mA cm^–2^, each as a function of pH (Figure 3b). The linear pH-dependent relationship *E*_a,p_ vs pH leads to a slope of −89 mV per pH unit, much higher than the theoretical value of −59 mV per pH unit.^33^ The latter is interpreted as the oxidation of Ni^2+^(OH)_2_ to Ni^3+^O(OH) with 1H^+^/1e^–^ transfer, but the actual slope is nearly 1.5 times higher than the theoretical value. This corresponds to a 3H^+^/2e^–^ coupled oxidation process.^27,33^ Furthermore, the RHE scale is used to determine the reaction order with respect to pH, and to prevent the contributions of thermodynamic driving force changes.^34^ In the case of an OER process following a prototypical concerted proton–electron transfer (c-PET) route, the reaction order in pH (on the RHE scale) is theoretically known to be zero.^34−36^Figure 3c shows negligible pH-dependent behaviors at 1.60, 1.63, and 1.66 V vs RHE, indicating a c-PET process involved during the OER. Therefore, proton transfer plays a crucial role in water oxidation over the Ni-based catalysts. It is worth noting that the reaction order of pH increased with increased applied potential. This is likely caused by the limited catalytic activity observed under low pH stemming from nominal proton transfer, notwithstanding the fast electron transfer.^16^ Likewise, in the presence of organic anions, Ni-based catalysts exhibit negligible pH-dependent OER kinetics according to the obtained reaction order close to zero (Figures S9c, S10c, and S11c). Regardless, the correlation of the anodic oxidation peak with pH presents larger slope values when organic anions are introduced (Figures 3e, S9b, S10b, and S11b). This means that more protons are involved in the oxidation of Ni species if the electron transfer number remains constant. Further, the injection of organic anions positively shifts the oxidation peak despite an increase in pH, contrary to the result of a single pH effect. This interesting trend could be ascribed to the presence of organic anions in the interlayer space of metal hydroxides. Simply put, the carboxylates are likely to impede the diffusion of hydroxide ions from the bulk electrolytes into the interlayer, thereby directly influencing deprotonation during Ni oxidation.^18,37^

FT-IR and GIXRD results confirm that organic anions can intercalate into the interlayer space of layered double hydroxides, specifically by replacing the pristine sulfate (more discussion is given in Figure S12). As shown in Figure S13a, *in situ* Raman spectroscopy at 0.593 V vs Hg/HgO demonstrates the surface-adsorbed (uncoordinated) carboxylate through a peak at ∼1640 cm^–1^. This indicates that the introduced organic ligands do not form coordination bonds with the metal hydroxides but are electrostatically adsorbed on the surface.^11,35^ This result is reasonable since carboxylates are strongly electron-donating in nature as ligands and coordinate to Ni^2+^ (located at the borderline of hard and soft acids)^38^ to generate coordination compounds that exhibit a limited alkaline hydrolysis resistance, as confirmed in our previous work.^11^ Indeed, all experiments were conducted using a rotating disk electrode, wherein diffusion limitations can be greatly reduced under a high rotation speed. Figure S14 reveals that the Ni catalyst exhibited higher OER current density at 1600 rpm than that at 0 rpm, an observation consistent with higher OH^–^ availability under elevated rotation. However, the oxidation peak potentials were identified to be identical under high speed and without rotation. This suggests that the OH^–^ content in the catalyst interlayer is minimally impacted by the RDE speed in the same pH solution. Besides, the same oxidation peak trends are observed with positive shifts, whereas unchanged OER polarization currents can be identified at both 0 and 1600 rpm after adding the organic anions. These characterization data led us to speculate that the Ni redox reaction potentials are primarily dominated by anions in the interlayer space, such as OH^–^ and guest anions.^18,19,39^ The oxidation peak shifts likely occur from the insertion of organic anions into the interlayer spaces, causing a drop of OH^–^ ions (consistent with Coulomb’s law of electrostatic interactions). This explains why adding BTC^3–^ causes a higher positive shift of the oxidation peak. To this end, most experimental studies and theoretical calculations are based on the edge of Ni or NiFe hydroxides as the active site locations. In contrast, deprotonation during oxidation is known to be facilitated owing to the low OH^–^ diffusion barriers.^40,41^ This is why, under concentrated alkaline media, the diffusion limitation is not dominant for the OER polarization curve.

**Figure 4:**
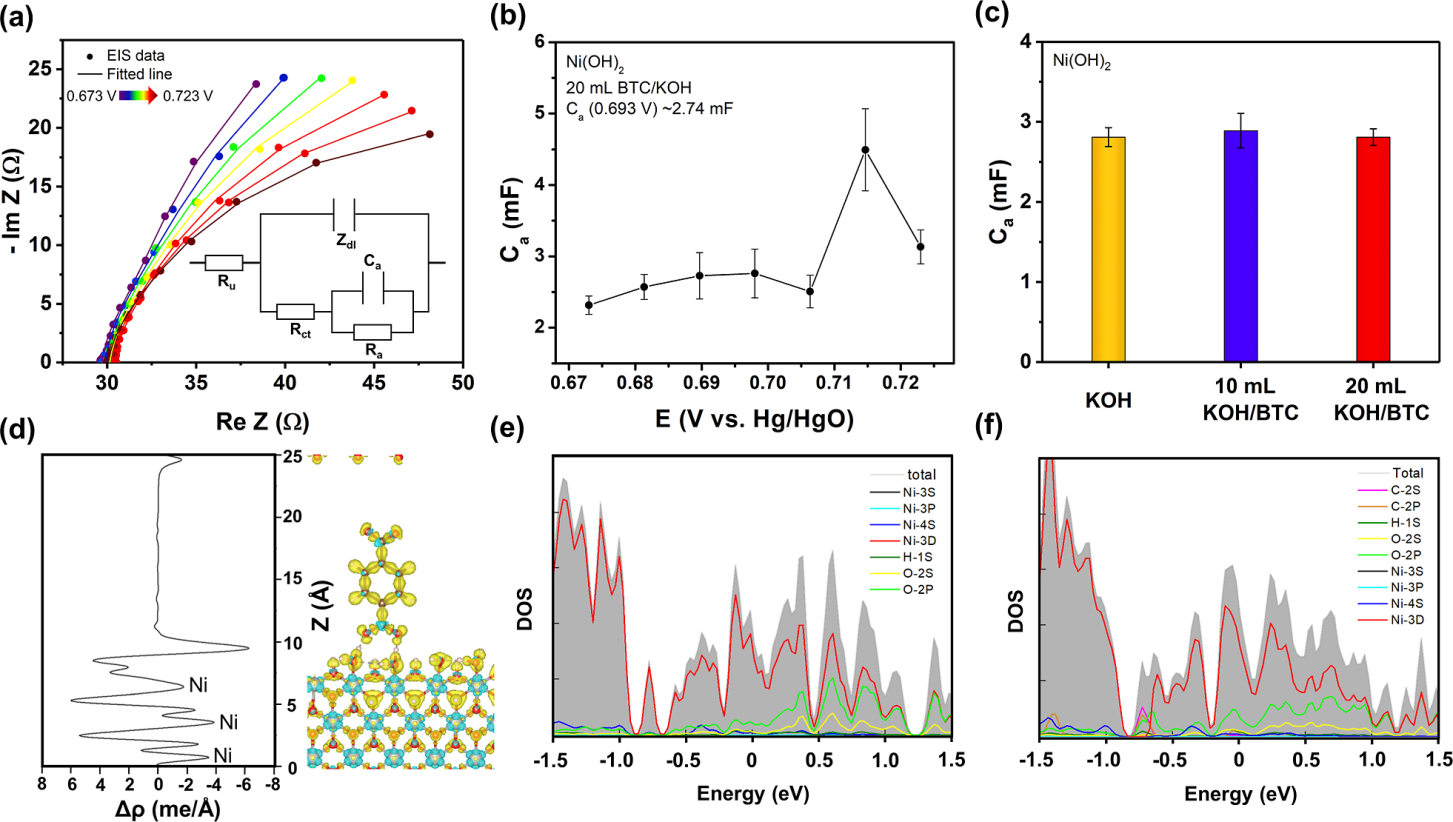
(a) Electrochemical impedance spectroscopy (EIS) recorded from 0.673 to 0.723 V vs Hg/HgO. All data were fitted according to the equivalent electric circuit in the inset. It includes an electrolyte resistance (*R*_s_), a double layer impedance (*Z*_dl_), a charge transfer resistance (*R*_ct_), an adsorption capacitance (*C*_a_), and an adsorption resistance (*R*_a_). (b) Correlation of the adsorption capacitance with the potential in the presence of BTC^3–^. (c) Comparison of adsorption capacitances (obtained at 0.693 V vs Hg/HgO) across the addition of BTC^3–^ into the electrolyte. (d) Charge densities of surface-hydroxylated NiOOH in the presence of BDC^2–^. Yellow areas represent electron accumulation, blue areas represent electron depletion. The plane-averaged charge density difference along *z* direction is shown on the left panel. The density of states (e,f) of surface-hydroxylated NiOOH surfaces, without and with BDC^2–^.

Relying upon EIS, adsorption capacitance is recognized as a metric to accurately estimate the real electroactive surface areas for metal oxide OER catalysts.^42,43^ To further augment the effect of organic anions on the OER activity, the Ni(OH)_2_ electrode was subjected to a series of EIS experiments (Figure 4a and Table S2). The adsorption capacitances were obtained by fitting Nyquist plots, predicated upon the Armstrong–Henderson equivalent electric circuit.^44^ Our previous work demonstrated that nearly all the active sites in NiO_*x*_ become active at 1.60 V vs RHE (0.693 V vs Hg/HgO), and the adsorption capacitance usually reaches a plateau.^42^ In Figure 4b, the Ni catalyst shows an adsorption capacitance of around 2.74 mF at 0.693 V vs Hg/HgO. After injecting BTC^3–^ into the electrolyte, similar adsorption capacitances were obtained (Figure 4c). This result suggests that the carboxylates in the electrolyte do not block the electrocatalytically active sites or do not increase the number of sites.

DFT calculations were performed to elucidate the influence of organic anions on the Ni catalysts. In our study, supercell modes for the NiOOH nanosheet were established. Figures 4d and S16 show surface-hydroxylated NiOOH in the presence or absence of BDC^2–^ adsorption. It is evident that in both systems, Ni atoms reside in the charge depletion region, and the other atoms assume the charge accumulation region. To further confirm the charge visually, we calculated the plane-averaged charge density difference (Δρ) along the *z* direction, given by Δρ = ρ_total_ – ρ_NiOOH_ – ρ_BDC_, where ρ_total_, ρ_NiOOH_, and ρ_BDC_ represent the plane-averaged charge densities of the adsorption system, isolated NiOOH, and BDC, respectively. Here, Δρ > 0 indicates electron accumulation, while Δρ < 0 indicates electron depletion. Figure 4d (left panel) shows that Ni atoms reside in the charge depletion region; in turn, O atoms are in the region of electron accumulation. Similar charge density distribution profiles indicate that the addition of the BDC^2–^ anion has no effect on the catalytic interface. The density of states (Figure 4e,f) further suggests that the introduction of BDC^2–^ also has a negligible contribution to the electrocatalyst near the Fermi energy level.

## Conclusions

In summary, the reconstruction of MOFs as a synthetic route to afford exceptionally active metal hydroxide catalysts has been introduced previously, and based on the observations, this method merits further critical interrogation. As one of the most essential components in MOFs, organic ligands leach into the electrolyte during alkaline hydrolysis, and their role largely remains a matter of conjecture. Here, we examined the effects of disparate carboxylates in electrolytes on the OER of electrodeposited metal hydroxides as a reference system after excluding one or more of the other variables, such as electrolyte purity, ohmic loss, electrolyte pH, and rotational speed, on a case-by-case basis. Our results indicate that the introduction of organic anions into the alkaline electrolyte did not affect the OER activities of LDH-type Ni or NiFe-based hydroxides, yet it significantly altered the redox potential of the Ni species. This is because the organic anion can insert into the interlayer space of the layered metal hydroxides, thus affecting the deprotonation rate accompanying the oxidation of the Ni species. However, the electrostatically adsorbed organic anions at the edge of the nanosheet form weak noncovalent interactions with the active sites through hydrogen bonding. Due to the low diffusion barrier at the edges, the presence of organic ligands has not been found to alter the catalytic activity in strong alkaline solutions. Our work offers a good reference for custom-designed electrolyte components and the study of electrolyte anion/cation effects. In particular, research interest in the addition of new constituents to the electrolyte should be concerned not only with their effect on the catalytic activities but also with the variation of system parameters that accompany their introduction. It is high time for future MOF metamorphosis research to focus on the coordination modes and coordination strengths offered by a plethora of organic ligands with low-cost transition metal nodes, as well as to study their influences on the rates of structural derivatization, rearrangement of metal clusters, and connection modes thereof, eventually leading to the derived metal hydroxide phase mixtures.

## Supporting Information Available

Experimental section and characterization details, XRD patterns, ^1^H NMR spectra, Raman spectroscopy, FT-IR spectra, and RDE data, EIS fitting data, CV curve variations of the NiFe-LDH, pH-dependent CV curves, and ligand/KOH solution recipe (PDF)
