# Supplementary material for: Impact of Organic Anions on Metal Hydroxide Oxygen Evolution Catalysts
Source: ACS Catal. 2024 Jul 29;14(16):12074–81. doi: 10.1021/acscatal.4c01907 (PMC11334168; doi:10.1021/acscatal.4c01907)
Supplement: Supplementary file 1 — cs4c01907_si_001.pdf [file cs4c01907_si_001.pdf]

Supporting Information

Impact of organic anions on metal hydroxide oxygen evolution catalysts

Shujin Hou,[1,2,3,4] Lili Xu,[5] Soumya Mukherjee,[2,6] Jian Zhou,[1] Kun-Ting Song,[1] Zhenyu Zhou,[2,7] Shengli Zhang,[5] Xiaoxin Ma,[1,2] Julien Warnan,[2] Aliaksandr S. Bandarenka,[,1,8] Roland A. Fischer[*,2,8]*

1 Physics of Energy Conversion and Storage, School of Natural Sciences, Department of Physics, Technical University of Munich, James-Franck-Straße 1, 85748 Garching, Germany

2 Inorganic and Metal-Organic Chemistry, School of Natural Sciences, Department of Chemistry, Technical University of Munich, Lichtenbergstraße 4, 85748 Garching, Germany

3 Department of Chemistry and Biochemistry and the Oregon Center for Electrochemistry, University of Oregon, Eugene, OR, 97403, USA

4 Department of Chemical & Biomolecular Engineering, University of California, Berkeley, California, 94720, USA

5 Institute of Optoelectronics & Nanomaterials, College of Materials Science and Engineering, Nanjing University of Science and Technology, Nanjing, 210094 Jiangsu, China

6 Department of Chemical Sciences, Bernal Institute, University of Limerick, Limerick V94 T9PX, Ireland

7 School of Chemistry and Chemical Engineering, Nanchang University, Nanchang 330031, PR China

8 Catalysis Research Center, Technical University of Munich, Ernst-Otto-Fischer-Straße 1, 85748 Garching, Germany

* Corresponding authors:

Aliaksandr S. Bandarenka, email: bandarenka@ph.tum.de

Roland A. Fischer, email: roland.fischer@tum.de

Experimental section

Chemical reagents

Nickel(II) sulphate hexahydrate ($NiSO_4·6H_2O$, ≥99 %, ACS) and potassium hydroxide pellets (KOH, ≥85 %, Fe ≤0.0005 %) were purchased from Carl Roth GmbH & Co. KG (Germany). Benzene-1,4-dicarboxylic acid (H_2BDC, 98%, Sigma-Aldrich), nickel(II) chloride hexahydrate ($NiCl_2·6H_2O$, 99.3%, Alfa Aesar), iron(II) chloride tetrahydrate ($FeCl_2·4H_2O$, 99%, abcr GmbH & Co. KG, Germany) and absolute ethanol ((99.9%, Th. Geyer GmbH & Co. KG, Germany) were bought for the synthesis of Ni or NiFe surface mounted metal-organic frameworks (SURMOFs). Iron(II) sulfate heptahydrate ($FeSO_4·7H_2O$, ⩾99.0%, Sigma-Aldrich) was used to prepare the NiFe layered double hydroxides (LDH). Benzoic acid (BA, ⩾99.5%, Sigma-Aldrich), benzene-1,3,5-tricarboxylic acid (BTC, 98%, Thermo Fisher Scientific Inc.), pyromellitic acid (PMA, 96%, Alfa Aesar), and 2,3,5,6-tetrafluoro-1,4-benzenedicarboxylic acid (TFBDC, 97%, Sigma-Aldrich) were used for the investigations of organic anion effect. All reagents, except KOH, were used directly without purification. The commercial KOH was purified based on the method of Trotochaud et al.

Synthesis of SURMOFs and metal hydroxides

Ni or NiFe SURMOFs were prepared via a layer-by-layer (LBL) method on the modified gold electrodes. Please refer to our previous experiments for more synthesis details. Nickel SURMOF (60 cycles) is 82.5 μg and NiFe (Ni45- Fe15 layers) SURMOF is ~65 μg as measured by the quartz crystal microbalance (QCM). Both $Ni(OH)_2$ and NiFe-LDH were synthesized by a typical electrodeposition method. For $Ni(OH)_2$, 1.052 g $NiSO_4·6H_2O$ was dissolved into 100 mL fresh ultrapure water (18.2 MΩ·cm) with Ar (99.99%) purging over the solution for 15 min, followed by a galvanostatical electrodeposition on a gold disk (0.196 cm^2) at a current density of -0.25 mA cm^{-2} for 30 min protected by Ar above the liquid level. In the procedure of NiFe-LDH synthesis, 0.788 g $NiSO_4·6H_2O$ and 0.278 g $FeSO_4·7H_2O$ were firstly dissolved into 100 mL ultrapure water under the protection of Ar. Subsequently, a current density of -0.25 mA cm^{-2} was applied on the gold disk for 30 min. All prepared metal hydroxides were carefully rinsed by deionized water for three times and then dried by Ar following. The amount of NiFe-LDH formed was recorded as 101 μg by depositing on the gold QCM electrode (area 0.785 cm^2). Additionally, the electrodeposition of NiFe-LDH on the gold/Si wafer for comparison with MOF derivatives was conducted, according to our previous experiments.

Preparation of organic ligand anion/KOH solution

All organic ligands were dissolved in KOH solution to obtain concentrations of 0.1 M each. One hour of sonication and overnight resting were performed to ensure complete ligand dissolution. In the case of BDC/KOH, 0.508 g H_2BDC was added into 9 mL commercial or purified 1 M KOH and 21 mL deionized water to obtain 0.1 M BDC/KOH. The effects of both acid-base neutralization and dilution on the OH^- concentration are considered during the solution preparation. Please refer to Table S1 for more details. Prior to the injection of organic ligand anions into the test electrolyte, the pH values of the electrolyte in the electrolyzer and the as-prepared organic

ligand anion/KOH solution were adjusted with a pH meter by supplementing the low pH solution with concentrated KOH (1 M).

Electrochemical measurements

The organic anion effect was studied using a three-electrode setup, where a Pt mesh and Hg/HgO (1 M NaOH, Bio-Logic, France) electrode were adopted as the counter and reference electrodes, respectively, and the metal hydroxides coated gold disk electrode (5 mm in diameter) was used as working electrode. To obtain a steady-state chemical structure, all catalysts were electrochemically activated by 40 cyclic voltammogram (CV) cycles at a fast scan rate (20 mV s^{-1}) using O_2-saturated electrolyte. Subsequently, OER polarization curves were collected with a scan rate of 5 mV s^{-1} and a rotation speed of 1600 rpm. In addition, the potential correction of the reference electrode and the electrochemically active surface area (ECSA) determined by electrochemical impedance spectroscopy (EIS) were both referred to our previous reports.[1,2]

Material characterizations

Nuclear magnetic resonance (NMR) spectra were conducted on the Bruker Avance III-HD 500 NMR Spectrometer. In the NMR measurements, $Ni(OH)_2$ was deposited on gold substrate and a five-hour CV was run within a potential region of 0-0.7 V vs. Hg/HgO at scan rate of 50 mV s^{-1}. 0.1 M BDC/KOD was prepared by dissolving H_2BDC and KOH in heavy water (D_2O). Inductively coupled plasma atomic emission spectroscopy (700 Series ICP-OES by Agilent Technologies) was conducted to determine the contents of iron in commercial and purified KOH electrolytes. Infrared spectra of NiFe-LDH before and after organic anion effect studies were performed by a Fourier transform infrared spectrometer (FT-IR, PerkinElmer Frontier) with an attenuated total reflectance sampling accessory. The NiFe-LDH was electrodeposited on a gold/Si wafer. Grazing incidence X-ray diffraction (GIXRD) measurements for the catalyst thin-film were conducted on an X'Pert PRO PANanalytical instrument operating in a diffraction angle range of 5-90 degree. To determine the chemical state of organic anions at the electrode interface, *in-situ* Raman spectroscopy was acquired by the Renishaw inVia Reflex Raman microscope with a 532 nm laser source in a home-made cell.

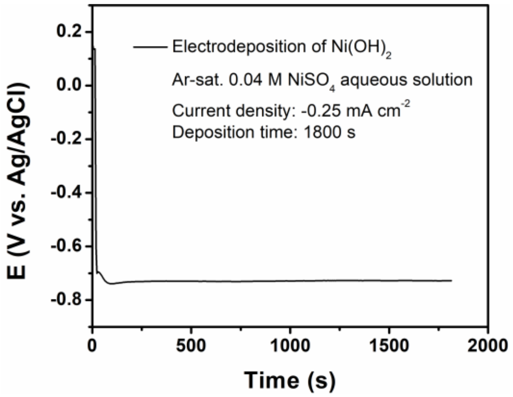

Figure S1. Electrodeposition of $Ni(OH)_2$ in Ar-saturated 0.04 M $NiSO_4$ solution, at a constant current density of -0.25 mA cm^{-2} for 1800 s.

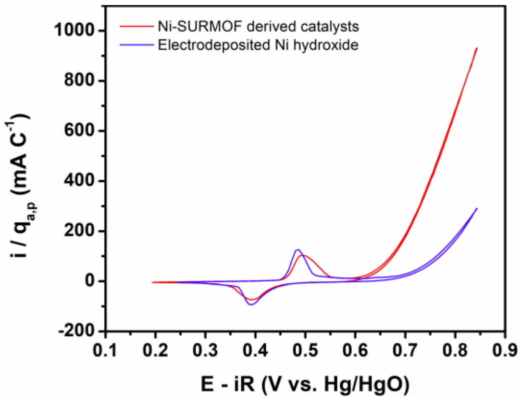

Figure S2. Charge-normalized polarization curves of Ni-SURMOF derivatives and electrodeposited Ni hydroxide, obtained by integrating the oxidation peak area. Data were extracted from the CV curves in Figure 1c (main text).

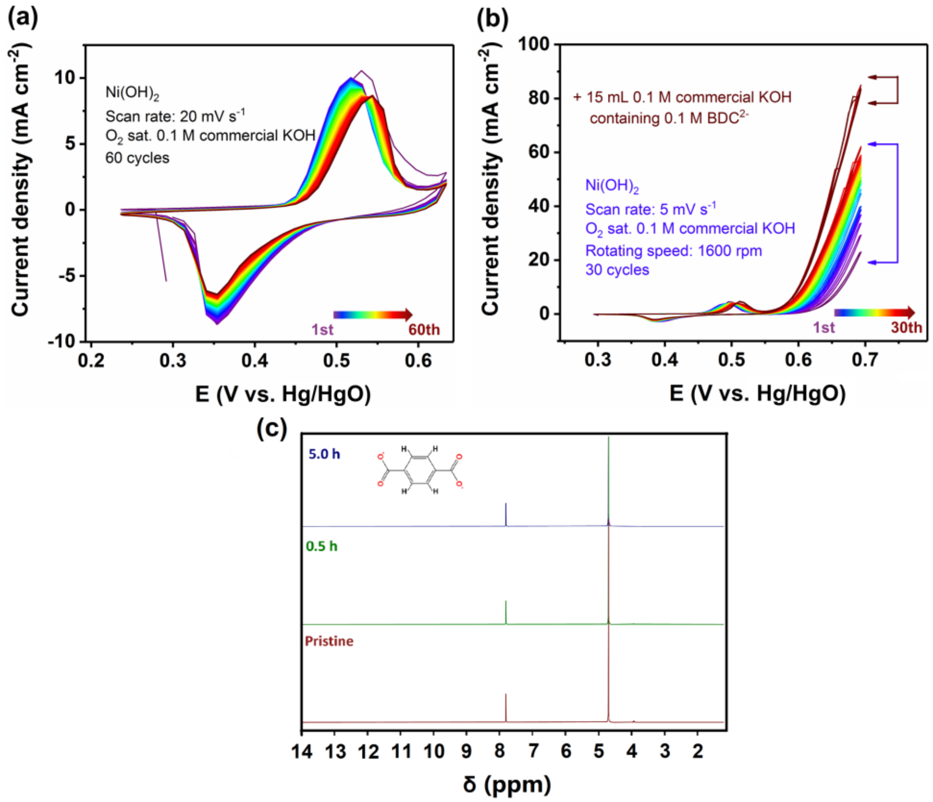

Figure S3. (a) CV curves recorded for Ni(OH)$_2$ thin film activation in O$_2$ saturated 0.1 M commercial KOH. (b) OER polarization curves in 0.1 M commercial KOH (blue arrow zone) and after the injection of 15 mL 0.1 M commercial KOH containing 0.1 M BDC^{2-} (brown arrow zone). (c) NMR spectra of the deuterated BDC^{2-}/KOD electrolyte before and after five hours of cyclic voltammetry. The cyclic voltammetry of deposited Ni(OH)$_2$ was performed with scan rate of 50 mV s^{-1} and a potential region of 0-0.7 V vs. Hg/HgO.

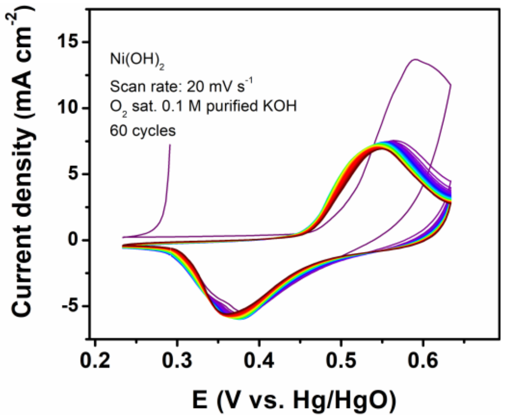

Figure S4. CV plot recorded at a scan rate of 20 mV s^{-1} for 60 cycles, accompanying the electrochemical activation of Ni(OH)$_2$ in O$_2$ saturated 0.1 M purified KOH.

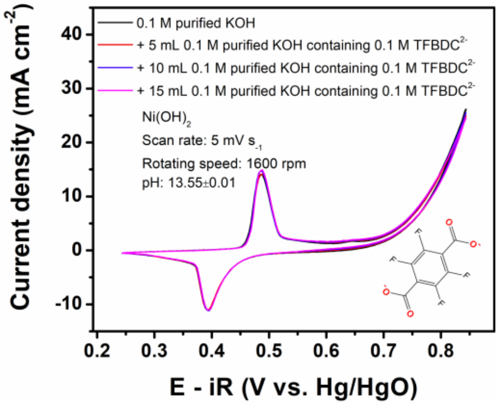

Figure S5. Studying the effect of adding TFBDC anions on the OER activity of $Ni(OH)_2$ catalyst. The experiment was conducted by recording OER polarization curves, after adding different volumes of $TFBDC^{2-}$.

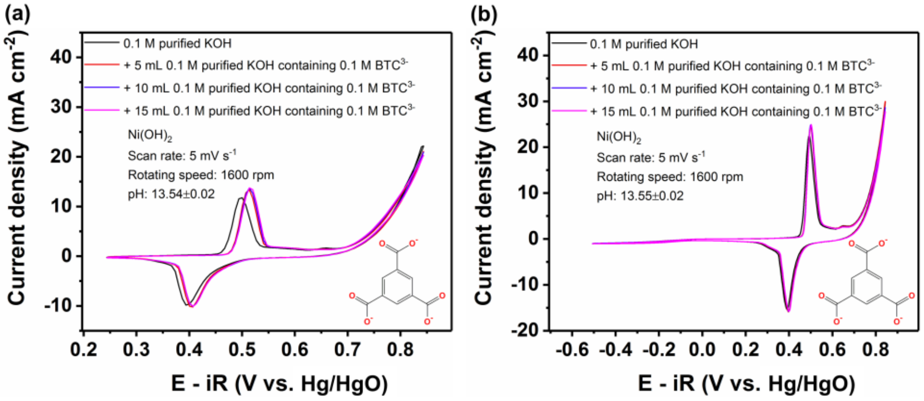

Figure S6. (a) Variations of OER polarization curves with the addition of different amounts of BTC^{3-}. (b) Polarization curves of $Ni(OH)_2$ performed across a wide potential range (-0.5 V to 0.793 V vs. Hg/HgO) in the presence of BTC^{3-}.

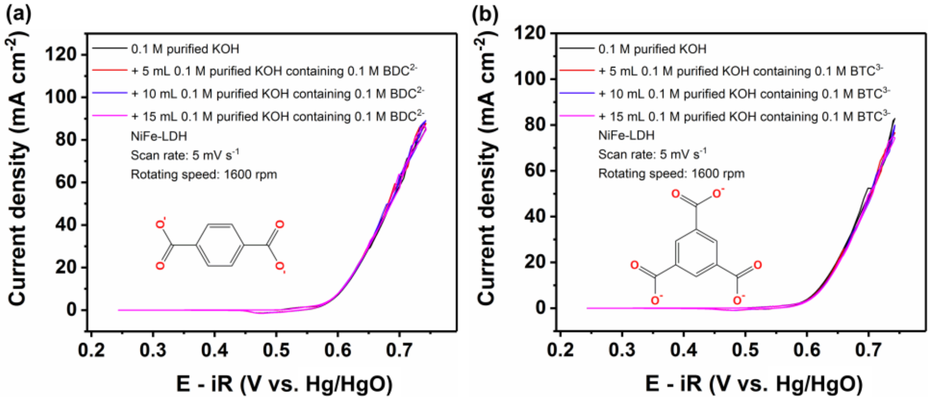

Figure S7. CV curve variations of the electrodeposited NiFe-LDH upon the addition of varying amounts of BDC^{2-} (a) and BTC^{3-} (b).

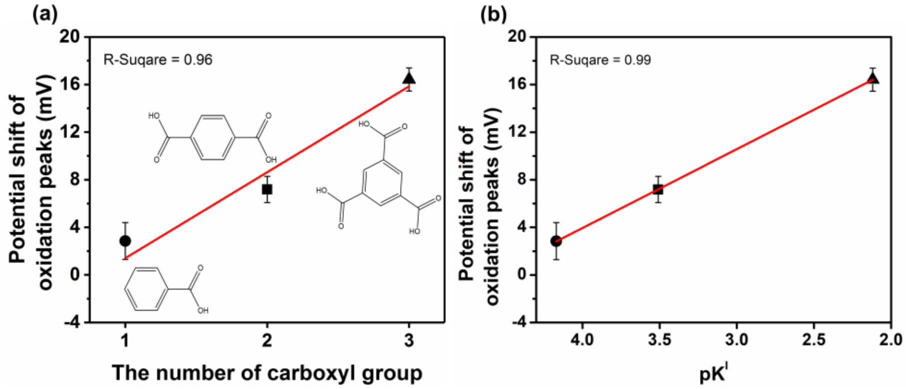

Figure S8. (a) Correlation between the number of ligand carboxylates and the potential shifts of oxidation peaks based on the OER polarization data plots. (b) Potential shifts of oxidation peaks as a function of first dissociation constants (pK^1) of the carboxylate ligands. The carboxyl group of BA is attached to an sp^2-hybridized carbon which is more electronegative and electron-withdrawing than the sp^3-hybridized carbon attached to the carboxyl group of acetic acid. The withdrawing inductive effects promote electron density away from the carboxylate anion, dispersing the charge, creating a stabilizing effect, and increasing the acidity of the carboxylic acid. When the H in the para position of benzoic acid is substituted with another carboxylic group, the carboxyl group is an electron withdrawing group, making the BDC higher pK^1. Likewise, the withdrawing inductive effects are further increased in BTC, corresponding to the highest pK^1. In turn, BTC^{3-} has the lowest ability to acquire protons, while deprotonation is the limiting step in the Ni oxidation process. Therefore, the redox properties that depend on the pKa of organic acids can be interpreted according to the ability to acquire protons.

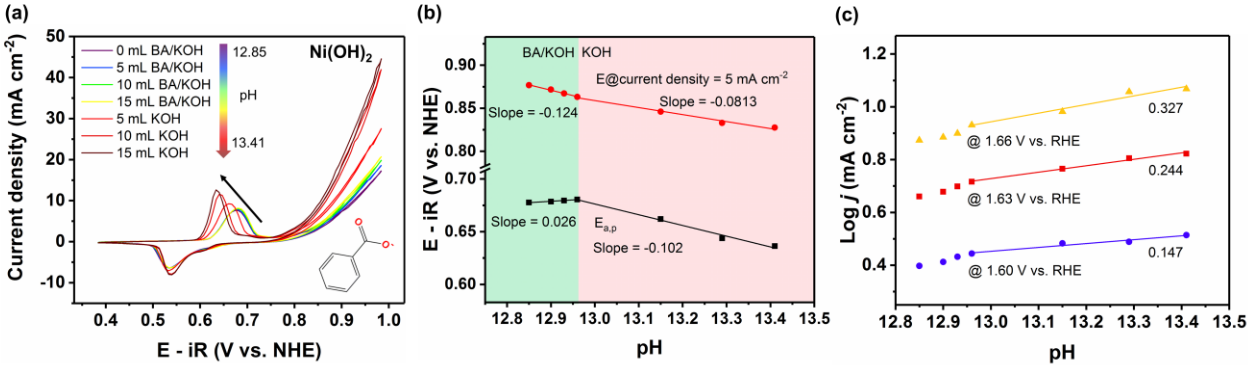

Figure S9. (a) pH-dependent CV curves of $Ni(OH)_2$ in the presence of BA^-. The initial electrolyte was prepared by mixing 10 mL 1 M purified KOH and 190 mL water, which was defined as 0 mL KOH, followed by the addition of different volumes of 0.1 M BA/KOH, or 1 M purified KOH solution. (b) Correlations of pH with the anodic oxidation peaks ($E_{a,p}$), and the potentials obtained under a current density of 5 mA cm^{-2}. (c) The reaction order of pH values with the OER activities at 1.60, 1.63, and 1.66 V vs. RHE.

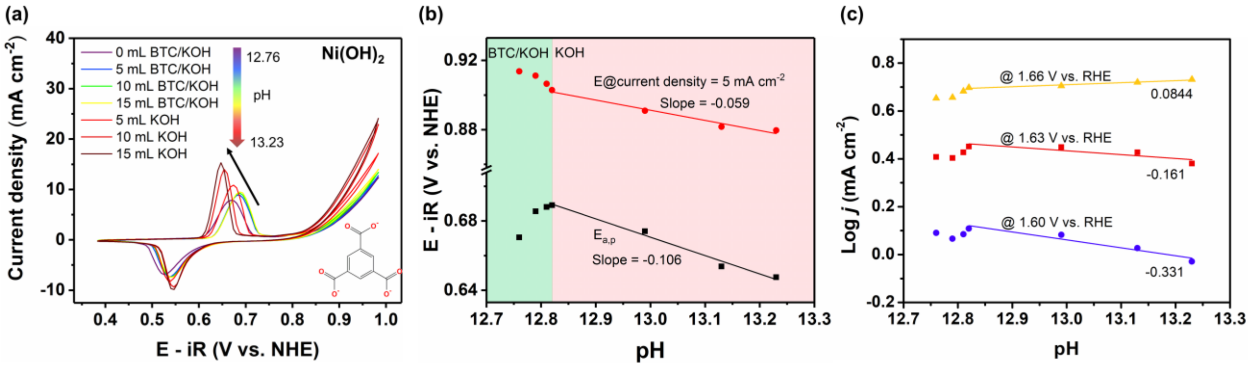

Figure S10. (a) pH-dependent CV curves of $Ni(OH)_2$ in the presence of BTC^{3-}. (b) Both anodic oxidation peaks ($E_{a,p}$) and potentials obtained at the current density of 5 mA cm^{-2} as a function of pH. (c) The reaction order of pH values with the OER activities at 1.60, 1.63, and 1.66 V vs. RHE in the presence of BTC^{3-}.

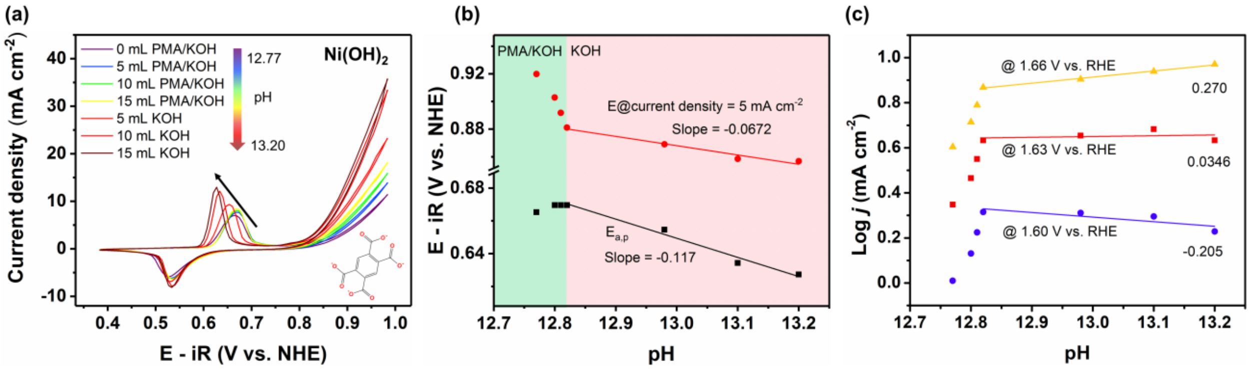

Figure S11. (a) pH-dependent CV curves of $Ni(OH)_2$ in the presence of PMA^{4-}. (b) Correlations of the pH with anodic oxidation peaks ($E_{a,p}$), and potentials obtained at the current density of 5 mA cm^{-2}. (c) The reaction order of pH values with the OER activities at 1.60, 1.63, and 1.66 V vs. RHE.

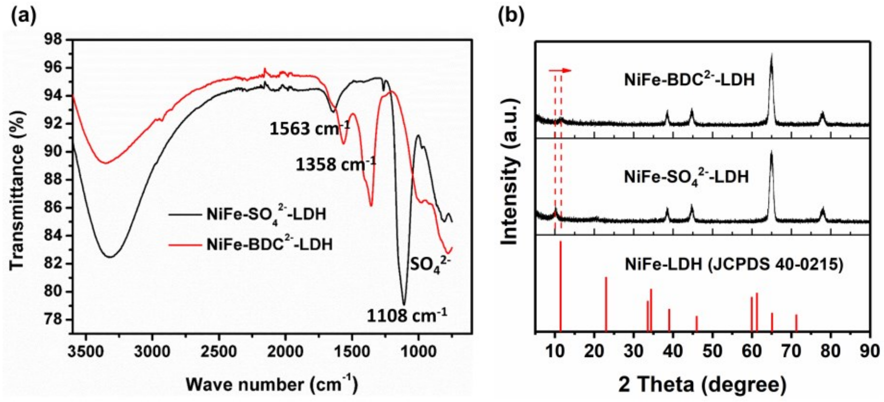

Figure S12. FT-IR (a) and GIXRD (b) measurements of the pristine NiFe-LDH and the NiFe-LDH cycled in BDC/KOH electrolyte. The pristine NiFe-LDH is denoted as NiFe-SO_4^{2-}-LDH since it was synthesized by using $NiSO_4$ and $FeSO_4$ as precursors. In the FT-IR spectra, the band at *ca.* 1108 cm^{-1} is assigned to $\nu_3(SO_4^{2-})$ mode in NiFe-SO_4^{2-}-LDH,[3] but it is found to disappear after CV cycling in the BDC/KOH electrolyte. Two new peaks at 1563 and 1358 cm^{-2} belong to the asymmetric stretching and symmetric stretching vibrations of the BDC^{2-} carboxyl groups.[1] In XRD data, the peaks appear at around 11.5°, corresponding to the (003) diffraction signal that can be used to determine the interlayer distance. It is noteworthy that this diffraction peak underwent a significant positive shift when NiFe-SO_4^{2-}-LDH was CV cycled in the BDC/KOH electrolyte, which can be attributed to the replacement of the interlayer SO_4^{2-} by BDC^{2-} and the reduction of the interlayer distance. All the above results confirm that interlayer SO_4^{2-} from NiFe-LDH could be replaced by BDC^{2-}, during the electrochemical measurements.

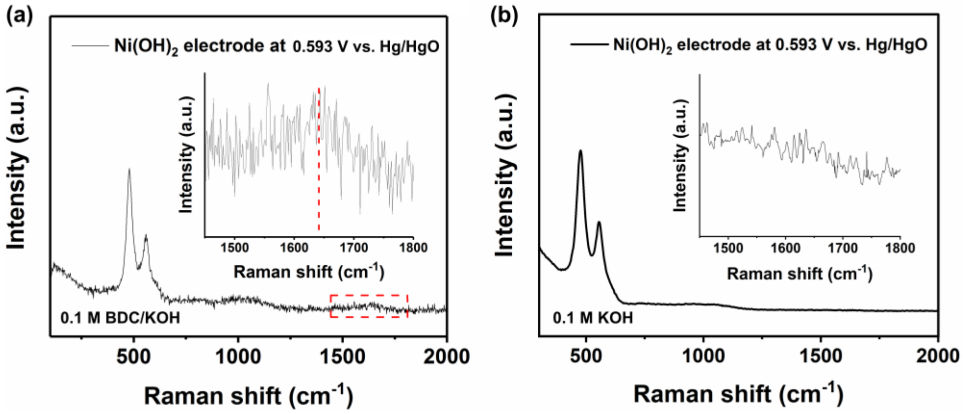

Figure S13. *In-situ* Raman spectrum of $Ni(OH)_2$ catalyst acquired at 0.593 V vs. Hg/HgO in 0.1 M BDC/KOH (a) and 0.1 M KOH (b).

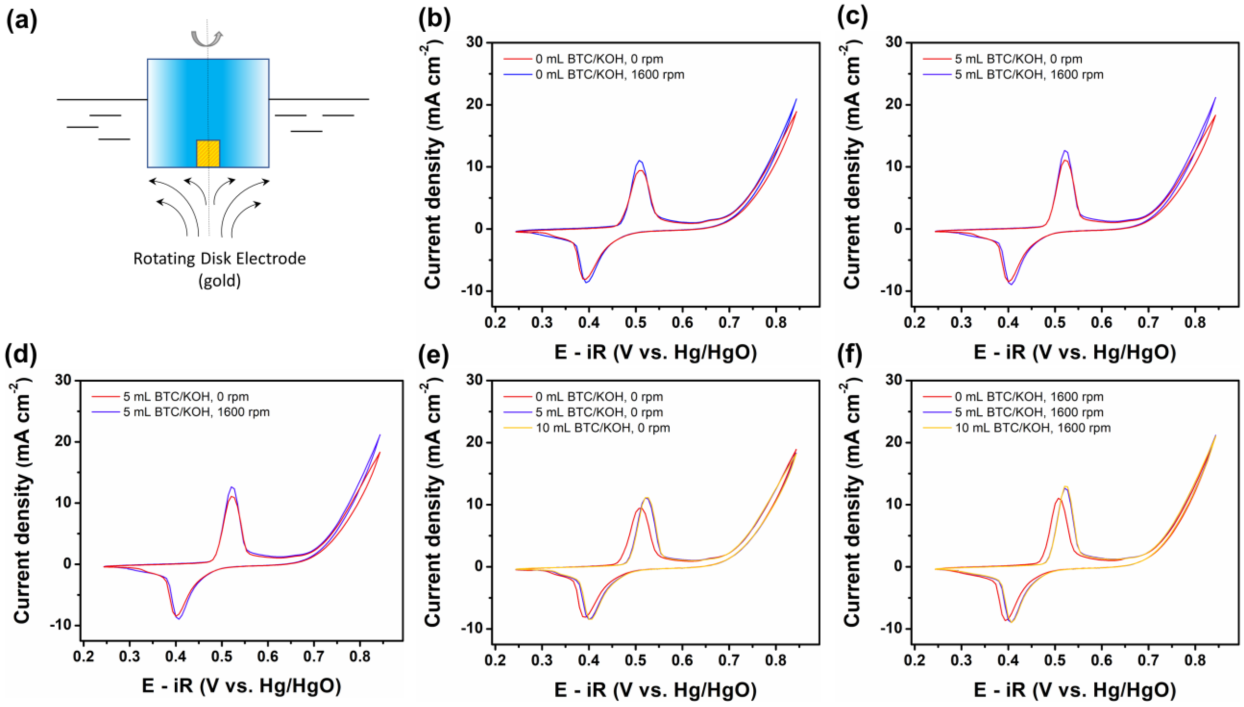

Figure S14. (a) Schematic illustration of a rotating disk electrode in electrolyte. (b-d) Rotation effect on the OER CV curves of $Ni(OH)_2$ in electrolytes spiked with 0, 5 and 10 mL of BTC/KOH. (e) Variations of OER polarization curves with the addition of different amounts of BTC^{3-} at 0 rpm. (f) The effect of BTC^{3-} amounts in electrolyte with a high rotation speed of 1600 rpm.

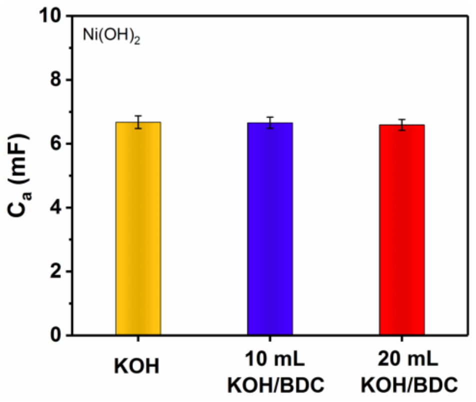

Figure S15. Adsorption capacitances of the $Ni(OH)_2$ electrode as a function of BDC amounts present in the electrolyte, recorded by applying a potential of 0.693 V vs. Hg/HgO in O_2-saturated 0.1 M purified KOH.

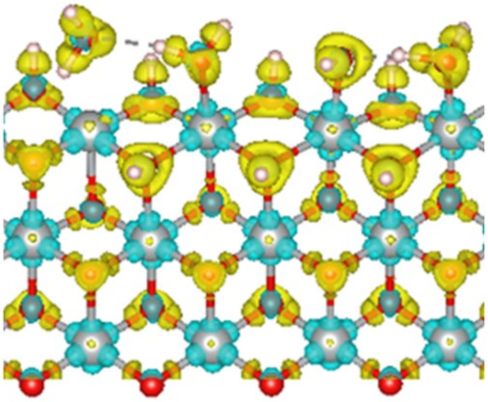

Figure S16. The charge densities of surface-hydroxylated NiOOH without BDC^{2-}.

Table S1. Preparation of various ligand/KOH solutions, each with specific carboxylic acid concentration and pH value. After preparing each solution, only a slight adjustment with KOH (1 M) is required to achieve the accurate pH value, based on the pH meter.

Concentration (M)	Ligand	Ligand Mass (mg)	1 M KOH (mL)	H_2O (mL)	pH
0.1	HBA	368.0	6	24	13.53±0.02
0.1	H_2BDC	508.0	9	21	13.55±0.01
0.1	H_3BTC	643.2	12	18	13.54±0.02
0.1	H_4PMA	778.0	15	15	13.54±0.01
0.1	H_2TFBDC	736.4	9	21	13.55±0.01

Table S2. Fitted values from the equivalent electric circuit in Figure 4a.

Potential (V vs. Hg/HgO)	R_u (Ω)	Z_{dl} (mF)	R_{ct} (Ω)	C_a (mF)	R_a (Ω)
0.673	29.72	4.02	14.6	2.51	57.51
0.681	29.99	3.95	20.11	2.57	67.17
0.690	30.12	3.78	19.01	2.61	50.52
0.698	30.16	3.67	19.86	2.95	39.63
0.706	30.27	3.39	16.29	2.76	35.67
0.715	30.45	3.47	19.29	3.20	26.48
0.723	30.11	3.17	16.90	3.42	24.91

References:

(1) Hou, S.; Li, W.; Watzele, S.; Kluge, R. M.; Xue, S.; Yin, S.; Jiang, X.; Döblinger, M.; Welle, A.; Garlyyev, B.; Koch, M.; Müller-Buschbaum, P.; Wöll, C.; Bandarenka, A. S.; Fischer, R. A. Metamorphosis of Heterostructured Surface-Mounted Metal–Organic Frameworks Yielding Record Oxygen Evolution Mass Activities. *Adv. Mater.* **2021**, *33* (38), 2103218.

(2) Watzele, S.; Hauenstein, P.; Liang, Y.; Xue, S.; Fichtner, J.; Garlyyev, B.; Scieszka, D.; Claudel, F.; Maillard, F.; Bandarenka, A. S. Determination of Electroactive Surface Area of Ni-, Co-, Fe-, and Ir-Based Oxide Electrocatalysts. *ACS Catal.* **2019**, *9* (10), 9222-9230.

(3) Jiang, X.-X.; Xue, J.-Y.; Zhao, Z.-Y.; Li, C.; Li, F.-L.; Cao, C.; Niu, Z.; Gu, H.-W.; Lang, J.-P. Ultrathin sulfate-intercalated NiFe-layered double hydroxide nanosheets for efficient electrocatalytic oxygen evolution. *RSC Adv.* **2020**, *10* (21), 12145-12150.
